# Application of Sensory Methods to Evaluate the Effectiveness of Solutions to Reduce the Exposure to Odour Nuisance and Ammonia Emissions from the Compost Heaps

**DOI:** 10.3390/s24134200

**Published:** 2024-06-28

**Authors:** Mirosław Szyłak-Szydłowski, Wojciech Kos

**Affiliations:** 1 Faculty of Building Services, Hydro and Environmental Engineering, Warsaw University of Technology, Nowowiejska 20 St., 00-653 Warsaw, Poland; wojciech.kos.dokt@pw.edu.pl; 2 Miejskie Przedsiębiorstwo Oczyszczania w M.St. Warszawie Sp. z o.o., Obozowa 43 St., 01-161 Warsaw, Poland

**Keywords:** compost, deodourisation, odours, olfactometry, sensory analysis, wastes

## Abstract

Exposure to high concentrations of odours can result in health effects associated with direct health risks and irritation from nuisance. This investigation aimed to correlate aspects of the waste composting process with the emission levels of malodourous compounds. An essential optimisation criterion is the reduction of negative environmental impacts, particularly odour emissions. This study characterises odour concentration variations across various technological variants over different weeks of the composting process. A secondary objective is evaluating the efficacy of these variants, which differ in inoculation substances and compost heap composition. Olfactometric analyses were conducted using portable field olfactometers, enabling precise dilutions by mixing contaminated and purified air. The primary aim was to examine the correlation between selected odour parameters, determined via sensory analysis, and ammonia concentration during different composting weeks. Ammonia levels were measured using an RAE electrochemical sensor. Research shows that odour concentration is a significant indicator of compost maturity. In situ, olfactometric testing can effectively monitor the aerobic stabilisation process alone or with other methods. The most effective technological solution was identified by combining olfactometric and ammonia measurements and monitoring composting parameters, ensuring minimal odour emissions and the safety of employees and nearby residents.

## 1. Introduction

The fundamental goal of waste management policy is to mitigate waste generation by tackling the root cause, reclaiming primary resources, repurposing trash, and ensuring the safe disposal of residual waste in the intermediate and final stages. The main objectives in this context are to optimise the recovery process while minimising any potential damage, including making necessary arrangements for ecologically safe storage. The latter procedure often occurs in mechanical–biological waste treatment facilities and compost plants, where waste disposal involves microorganisms in either an oxygenated or anaerobic environment.

This process is followed by releasing greenhouse gases (N_2_O, CO_2_, CH_4_) and odourants—volatile chemicals that cause odour [1]. These chemicals can disperse across significant distances. Depending on the prevailing weather conditions, the emitter’s range may extend to several kilometres. However, the assessment of smell resistance is typically conducted within a 500 m radius of the transmitter [2]. The ambient factor is of utmost significance in this context since it is linked to the perception of fishing chemicals around the odour source, resulting in olfactory distress [3,4]. Although the first element is not unpopular, the second component is a topic of much debate and uncertainty. The human nose is susceptible to even low quantities of odourants, including volatile organic compounds [5]. The perception of such chemicals involves many processes. The olfactory system does not respond to the presence of a specific odourant linearly concerning its concentration. Additionally, this response is somewhat subjective. However, odours are now classified as air pollutants and are subject to regulatory regulations in many countries [6,7]. Sironi et al. emphasise that these rules often pertain to the levels of smell irritation assessed by a panel of experts, but this approach needs to be revised [8]. This assessment quantifies the discomfort of those living near the encountered odour’s origin. It does not provide any details about the composition of the released particles, the intensity of the odour, or other related factors. Consequently, it is crucial to address the release of odour-causing substances in a manner consistent with other airborne contaminants. This involves creating olfactory analysis techniques and their incorporation into applicable legal regulations. Conducting olfactometric analysis and concentration investigations of chemicals is essential for studying the environmental effect of waste management facilities [7,9]. The process’s various stages are linked to gas composition alterations, resulting in changes in the quality and amount of odourants [10]. Furthermore, the release of certain chemical compounds is influenced by the transformations that occur throughout the four stages of aerobic biodegradation of waste: mesophilic, thermophilic, cooling, and maturation. The emission of aromatic compounds is altered in terms of both amount and composition as the waste input stream reaches the parameters of the following step.

The odourants produced during garbage’s mechanical and biological processing include volatile organic compounds such as terpenes [11,12]. While these chemicals may cause unpleasant odours, they are not toxic [13]. The emissions of volatile organic compounds from the compost plant, which consist primarily of hydrocarbons, p-limonene, ketones, and chlororganic compositions, may vary between 10 mg/m^3^ and 15 mg/m^3^ [14]. They may be generated by both oxygen processes and anaerobic methane fermentation [15,16]. Hydrolysis often breaks fatty acids into shorter-chain forms, such as acetic, propionic, and butyric acids. Furthermore, it is possible that one of the substances responsible for the smell in the vicinity of the compost business is hydrogen sulphide. This compound is created under settings when there is a lack of oxygen, such as poor ventilation in the compost pile [17]. Organic sulphates, also known as mercaptan, may be produced in both oxygen-rich and oxygen-deprived environments. However, when oxygen is present, they undergo a transformation into dimethyl sulphurs and dimethyl disars [18]. Terpenes, monocyclic arenas (benzenes C2, C3, and C4), alkanes, fluorine derivatives, and esters are some of the ecologically relevant chemicals created by waste’s biological processing [13].

During the process of composting, the breakdown of organic matter results in the release of readily degradable compounds, amines, and ammonia. The amount of these compounds emitted may range from 18 g per metric tonne to 1150 g per metric tonne of garbage [16]). The last component mentioned is present in the gas released from the compost plant at a concentration of up to 700 mg/m^3^ [19]. It is released during the enzymatic and microbiological anaerobic decomposition of proteins and amino acids [20].

The temperature of the process substantially impacts the release of volatile chemicals during composting. The saturation vapor pressure of the compounds found in biomass is increasingly dependent on the temperature. These effects are caused by the fact that these substances evaporate easily more quickly, become more concentrated in exhaled gases, and degrade more rapidly. This might result in a lack of oxygen, the activation of anaerobic microbes, and a shift in metabolic processes. Subsequently, chemically volatile substances, such as aldehydes and other carbonyl compounds, form, leading to increased odour irritation. 4,5-dimethyl-3-hydroxy-2(5H)-furanone is the compound that causes the characteristic odour known as the “hot compostage smell”. Inadequate process temperatures hinder the sterilisation of the biomass and impede the progress of the process.

The emission values of odourants from mature and fresh and ripe compost sites may vary from 5 ou/m^2^s to 150 ou/m^2^s [21,22]. Capelli et al. (2014) determined the odour emission variables for the compost factory. The green waste collecting process had the most significant geometric average OEF value of 3.02 × 10^5^ ou/Mg, with a median value of 3.30 × 10^5^ ou/Mg. On the other hand, the biological oxygen processes had the lowest OEF value of 3.99 × 10^6^ ou/Mg, with a median value of 2.99 × 10^6^ ou/Mg [23]. 

Although efforts have been made to standardise installations based on their resistance to odours and to align their effects for modelling reasons, each particular item should be approached separately, requiring inspections and field measurements to be conducted on each occasion. These items are sufficiently varied that predicted model computations cannot substitute for direct analysis [23]. Measurements of odours and volatile organic compounds are often conducted to examine the impact of gaseous pollutants on workers in large-scale MBP facilities, focusing on health impacts [24]. The presence of aromatic compounds in regions near MBP areas may be avoided or decreased using “pipe end” treatments. However, the workers working at these facilities are exposed to volatile organic compounds, ammonia, and hydrogen sulphide compounds [19,25,26,27].

Exposure to excessive odour may have adverse health implications in severe instances. Lower concentrations of odourants emitted in residential areas are associated with a range of health issues reported by those living nearby the source. They are not linked to a direct health concern [28] but are mostly associated with odour irritation, discomfort, and psychological consequences [29,30]. The residents living in the vicinity of the compost facility have many health issues, including nausea, diarrhea, extreme exhaustion, coordination problems, bronchitis, coughing in the morning, breathing difficulties, lethargy, eye irritation, frostbite, and allergies [31,32,33,34]. Furthermore, individuals residing within a distance of less than one kilometre to five kilometres from waste disposal sites and waste biodegradation plants experience unpleasant odours and various health issues. These include respiratory problems affecting both the upper and lower airways, irritation of the mucous membranes, allergies, a compromised immune system, skin disorders, deteriorating mood, gastrointestinal irritation, and cardiovascular complications [32,35,36,37,38,39,40,41]. 

Currently, injecting waste with mixes of microbes and enzymes is becoming more prevalent, alongside biofiltration, as an alternative to adding chemical compounds [42]. One notable approach among them is the use of EM biopreparations (effective microorganisms) and their variations. Teruo Higa developed the EM preparation by harnessing naturally occurring microorganisms found in the environment, such as lactic acid and photosynthetic bacteria, yeasts, radioactive organisms, and fungi [42,43].

Several factors, such as the rate at which bacteria consume oxygen and the breakdown of organic compounds during the latter stages of biostabilisation, influence the release of aromatic compounds.

Assessing the odour concentration fluctuation based on the biostabilisation cycle’s day is crucial. An intriguing research question is whether in situ olfactometric tests can effectively monitor and evaluate the progress of the oxygen stabilisation process of waste. Additionally, it is worth considering if these studies enhance the generally used offline optimisation approaches, which need sampling and time-intensive analysis of waste.

An attempt was made to correlate selected aspects of the composting process of waste and the emission levels of malodourous compounds accompanying these processes. An important optimisation criterion should also be minimising adverse environmental impacts, particularly the emission of odouriferous compounds. This paper, therefore, characterises changes in odour concentration values in various weeks of the composting process in different technological variants of this process. A partial objective, serving as a verification of the utility of the methodologies, is the evaluation of individual technological variants of the composting process, differing in the type of substances used to inoculate the waste heaps and the fraction content in the compost heap.

## 2. Materials and Methods

### 2.1. Description of the Research Object

The research was conducted in a composting facility that handles biodegradable trash, including desiccated foliage, mowed grass, and pulverised tree and plant limbs.

**Figure 1:**
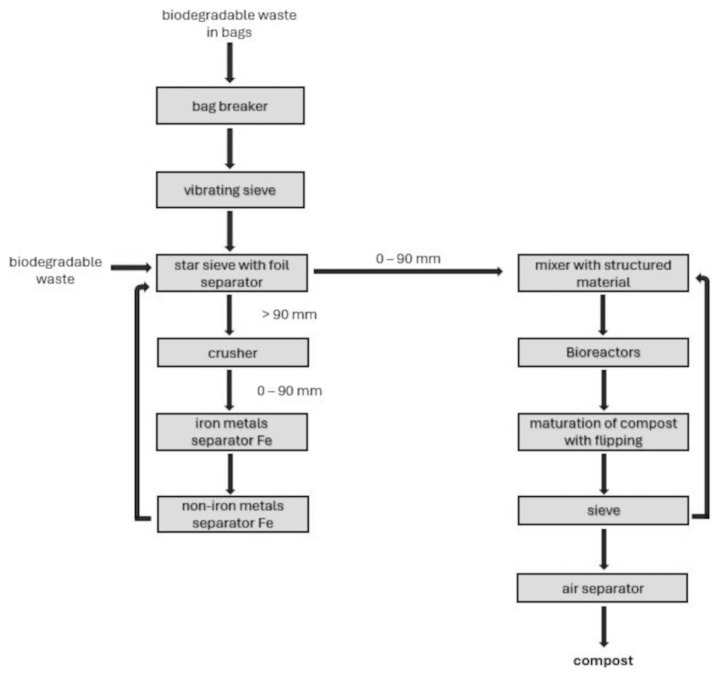
Block scheme for the management of biodegradable waste.

The garbage is processed at the compost plant using many steps, a cutting machine, vibrating and starry sieves, a crusher, and metal separators. The procedure also includes substance amalgamation, transference, and an air separator for screening and segregation. (Figure 1).

The composting method was carried out on a compost field at different process phases. Each prism had a trapezoidal shape, with dimensions of 4.5 m wide, 35 m in length, and 2 m in height. The waste mass at the prisms ranged from around 300 to 350 tonnes. Vehicles, such as wheel chargers, promote air circulation across their whole volume. The compost underwent a maturation period that lasted for 10 to 14 weeks.

### 2.2. Examined Variants

Examinations were conducted from August to October 2023, twice a week. The variants differed in the share of the intermediate fraction (with a diameter of less than 80 mm) and the addition of the preparation. The intermediate fraction refers to the material from sieving green waste with a three-faction sieve. This sieve classifies the trash into three categories based on size: less than 10 mm, 10 to 60–90 mm, and over 60–90 mm. An intermediate fraction falls within the size range of 10 to 60–90 mm.

Table 1 lists the variants analysed, differing in the proportion of the intermediate fraction and the type of preparation used. 

### 2.3. Characteristics of the Used Preparations 

Preparation A—a liquid that is a mixture of bacterial cultures that break down cellulose and limit the emission of ammonia and hydrogen sulfide compounds. It works in a wide range of pH oxygenic and anaerobic conditions. Dosage for a single prism: three doses of 6 L of the preparation diluted in 600 L of water. One dose—after dosage, two doses—after transplanting the prism.

Preparation B—a powder that is a mixture of bacteria, enzymes and roast-saline extracts. It supports technological processes and accelerates organic matter’s degradation, eliminating unpleasant odours accompanying waste processes. It has lymphatic properties. Dosage of the preparation: even distribution of the powder on the prism.

### 2.4. Gas Sampling

The static chamber used for testing was constructed from an odourless substance and had the form of a cylinder topped by a semicircular detruncated with a radius of 20 cm. The object has a height of 30 cm and is equipped with a nozzle to which the measuring tube can be connected. The active volumes, which refer to the volume of the air column above the tested surface, of the static chamber were measured to be 0.040 m^3^. 

**Figure 2:**
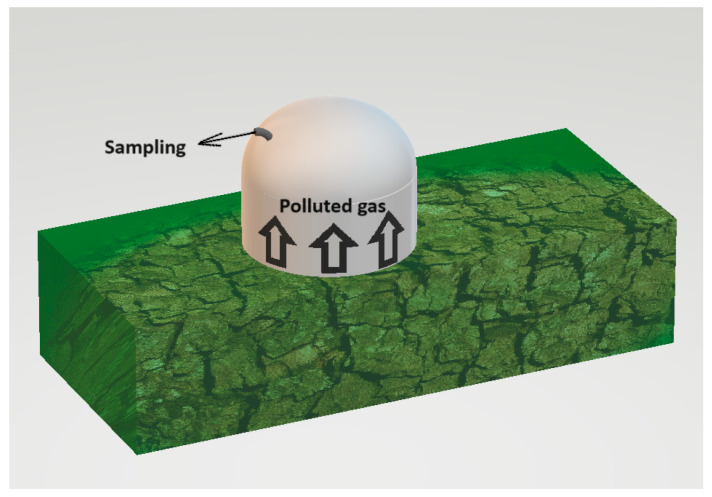
Sampling of the polluted air from the compost pile.

The presence of background odour while sampling is a significant constraint for assessing odour concentration. The research used stainless steel equipment, which is odourless and can be readily decontaminated. Exclusively virgin polytetrafluoroethylene (PTFE) tubing was used for both the collection of samples and the transportation of uncontaminated air. In addition, a carbon filter was used to purify the technical air, ensuring it was completely free of odours and preventing any background smells. Furthermore, the term “background odour” refers to the scent originating from the location of the sample. The evaluated techniques rely on segregating a segment of the contaminated air and quantifying the concentration of odour at the exit. It is essential to mention that the sample process separates the background odours. The detection threshold of this sort of sampling may be lower than the background odours already present in the environment [44]. The sampling method is presented in Figure 2. 

### 2.5. Odour Measurement

The efficacy of the deodourisation procedure was evaluated using a field olfactometry approach. The experiment used a Scentroid SM100 olfactometer and a static chamber for air sampling. Olfactometric measurements were conducted using portable field olfactometers, enabling the performance of a calibrated sequence of dilutions by combining polluted, odourous air with clean, filtered air. The concentration of odour is measured in odour units (ou) per unit volume (ou/m^3^) according to the European standard PN-EN 13725:2022 [45]. The Scentroid SM 100 is a portable olfactometer that can measure odour concentrations ranging from 2 to 30,000 ou/m^3^ [44]. The Nasal Ranger field olfactometer is often used; however, it has lower accuracy and range of determinability than this alternative [46]. Before each examination, the testers’ olfactory performance was assessed using a Sniffin’ Sticks test (SST) per the ISO 13301:2018 standard [47]. At each site, four olfactometric measurements were conducted. The smell concentration value is calculated as the geometric mean of the individual measurement findings [48].

### 2.6. Measurement of Ammonia, Temperature, and Humidity

Analyses were conducted using a special gas detector to detect ammonia emissions from compost prisms. The detectors were strategically positioned at the compost prisms to allow for precise monitoring of the content and concentration of the tested gas in the surrounding atmosphere. A systematic measurement trial was carried out over a period of time to gather reliable information on ammonia emissions from composting processes.

Ammonia was measured by a MultiRAE detector (RAE Systems, Honeywell, San Jose, CA, USA). The range of the electrochemical sensor was between 0 and 100 ppm, with a resolution of 1 ppm and a temperature range from −20 °C to 40 °C. Humidity inside the composting prism was measured using a compost hygrometer PMS 710 (Tsingtao Toky Instruments Co., Ltd., Qingdao, China) with accuracy ± 2% (non-saturation condition). Dimensions of the main unit: 140 mm × 60 mm × 22 mm, length of the needle: 280 mm. It was widely disseminated throughout various locations inside the compost prisms. This enabled monitoring variations in moisture during the composting process and evaluating whether optimal conditions existed for the effective breakdown of organic matter.

The temperature was assessed with a TROL2 compost thermometer (Dramiński Technology Co., Sząburg, Poland). Measuring range: −55–125 °C, resolution: 0.1 C, measurement accuracy ± 0.5 °C in the range from 0 °C to 85 °C, length of the needle: 300 mm. The thermometer is a lengthy instrument with a metal sensor inserted deep into the compost prisms. Accurate temperature measurement was critical for assessing the activity of microorganisms involved in breaking down organic materials and maintaining ideal conditions for the composting process. 

Concurrent humidity and temperature measurements were conducted alongside ammonia emission measurements, enabling a thorough assessment of environmental conditions throughout the composting process.

### 2.7. Statistical Methods

To compare nine datasets to determine which set differs the most, analysis of variance (ANOVA) was employed. Assumptions were checked using Levene’s test and the Shapiro–Wilk test. In cases where assumptions were not met, the Kruskal–Wallis test was conducted along with pairwise comparisons using Dunnett’s C Simultaneous Confidence Formula (DSCF). This method serves as a pairwise comparison technique in a one-way analysis of variance. It acts as a post hoc method, allowing for comparison of each group to the control (or a selected reference group) to determine which groups differ significantly. The DSCF approach relies on establishing confidence intervals for the differences between the means of individual groups and the mean of the control (or reference) group. This allows us to ascertain whether the differences between groups are statistically significant. The method utilises simultaneous confidence intervals, thereby addressing the issue of multiple comparisons and minimising the risk of Type I error (rejecting the true null hypothesis).

The steps in the DSCF method include:

1. Conducting one-way ANOVA to determine statistically significant differences between groups.
2. If the ANOVA test is statistically significant, a DSCF analysis for pairwise comparisons should be conducted.
3. Calculating the differences between group means and the mean of the control (or reference) group.
4. Establishing confidence intervals for these differences.
5. Checking if the confidence intervals do not contain zero. If the confidence intervals do not include zero, it indicates that the differences between groups are statistically significant.

An ANCOVA test was also performed, which can be applied even when the variances of dependent variables are not homogeneous; ANCOVA is less sensitive to this assumption than traditional one-way analysis of variance (ANOVA). ANCOVA can increase the study’s statistical power, even when variances are homogeneous, by controlling for a covariate variable that may affect the dependent variable. Moreover, even if variances are homogeneous, ANCOVA can still account for differences between groups in the means of dependent variables, which may be significant from a scientific or practical standpoint. The ANCOVA test was conducted with Tukey’s correction, specific to post hoc tests in ANOVA, which controls the significance level for all possible pairwise group comparisons. It allows for identifying particular differences between groups while simultaneously maintaining the risk of Type I error.

The Spearman coefficient was calculated to determine the strength and direction of the relationship between variables. Unlike the Pearson coefficient, the Spearman coefficient measures the degree of association between two variables but does not assume a linear relationship or normal data distribution. Instead, the Spearman coefficient is based on the ranks of observations, making it more resistant to outliers and more flexible in detecting non-linear relationships between variables. Additionally, partial correlation was examined, measuring the strength and direction of the relationship between two variables after controlling for the influence of other covariate variables. 

### 2.8. Methodology

Tests were performed on piles located on the composting plant. The interval between the series was from 3 to 7 days, and the complete cycle lasted a maximum of 14.5 weeks, starting from the dump of the pile until the composting process was completed. Samples were taken from five places on the plateau of the piles (corners and geometric centre). Sampling of air emitted from the surface sources of odourants was carried out as follows:

- From the height of 1.5 m above the plateau of the pile;
- Using static chamber from the surface of the pile;
- From a depth of 10 cm.

For each test, 10 min was required to equalise pressure from the time the static chamber was set. The total number of tested samples was 648.

## 3. Results and Discussion

### 3.1. Comparison of Research Variants

**Figure 3:**
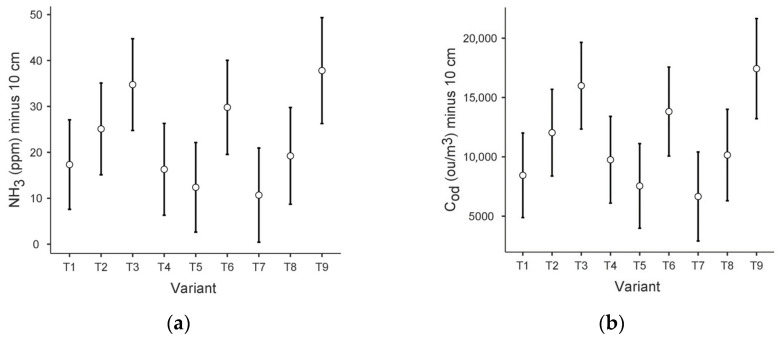
Plots of estimated boundary averages for the different variants: (**a**) ammonia concentration at −10 cm; (**b**) odour concentration at −10 cm.

The data sets obtained did not meet the assumptions of the ANOVA test (for Levene’s homogeneity test and the Shapiro–Wilk normality test *p* < 0.001). Therefore, the test that does not require homogeneity of variance—Kruskal–Wallis—was applied, with the grouping variable: variant and dependent variables: ammonia concentration scores and odour concentration scores at each level concerning the area of the prism (−10 cm, 0 cm, 1.5 m). Plots of the estimated boundary averages of ammonia (NH_3_) and odour concentration (C_od_) at the −10 level, with the component variant, are included in Figure 3. 

Significant statistical differences were observed for NH3 at the −10 level (*p* < 0.01). For NH_3_ at 0 cm and NH_3_ at 1.5 m, the significance level was 0.05 and 0.49, respectively). 

Regarding odour concentration results, significant statistical differences were also observed between the results obtained at a depth of −10 cm (*p* < 0.001). For the 0 cm and 1.5 m levels, the significance level was 0.08 and 0.63, respectively. 

The Dunnett’s C Simultaneous Confidence Formula (DSCF) pairwise comparison used showed that significant statistical differences (significance level *p* = 0.01) occurred for the following pairs for both NH_3_ and C_od_ at 10 cm depth: T3 and T5, T3 and T7, T5 and T6, T5 and T9, T6 and T7, T7 and T9. 

These results were confirmed via the ANCOVA test without correction, while the application of the Tukey correction excluded, among the above set, significant differences (significance level *p* > 0.05) for pairs T3 and T5, T5 and T6 and T6 and T7. 

### 3.2. Correlations between Variables

**Figure 4:**
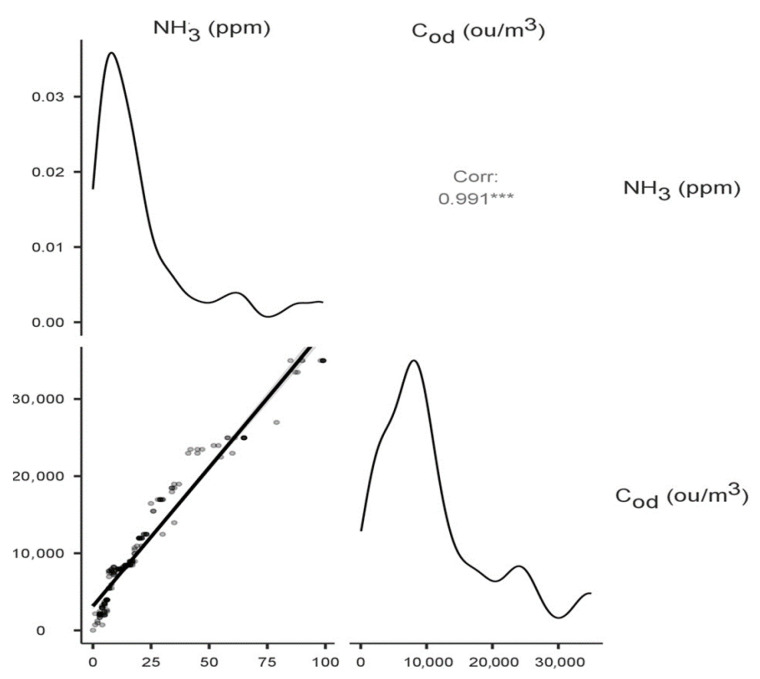
Correlation matrix with density plot for the variables: ammonia concentration—odour concentration at −10 cm. The asterisks next to the correlation coefficient indicate the value of the test probability.

The highest statistically significant (*p* < 0.001) values of correlation coefficients between variables were recorded for NH_3_ at −10 cm and NH_3_ at 0 cm (Spearman’s rho correlation coefficient 0.551), NH_3_ at 1.5 m (0.434), and C_od_ at −10 cm (0.991), C_od_ at 0 cm (0.533) and C_od_ at 1.5 m (0.410). No statistically significant correlations were recorded for the determined concentrations and heap temperature and humidity values. Figure 4 shows a matrix plot with a scatterplot, density plots of the individual variables, and a correlation factor for the variables: ammonia concentration—odour concentration at −10 cm (the highest correlation coefficient). The density curves show how the data in each variable are distributed.

**Figure 5:**
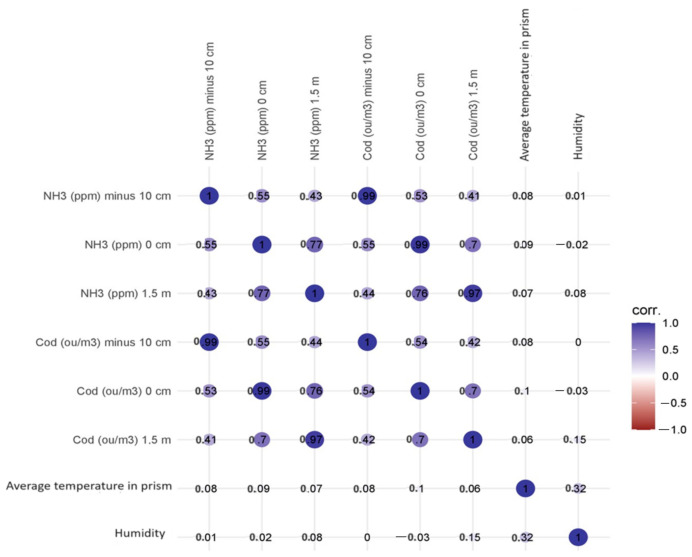
Correlation matrix of examined parameters.

The values of the correlation coefficients did not change significantly when the control variables were mean heap temperature and heap humidity; they increased from 0.001 to 0.0.004. The correlation matrix can be found in Figure 5. 

### 3.3. Comparison of Results by Week of a Study

**Figure 6:**
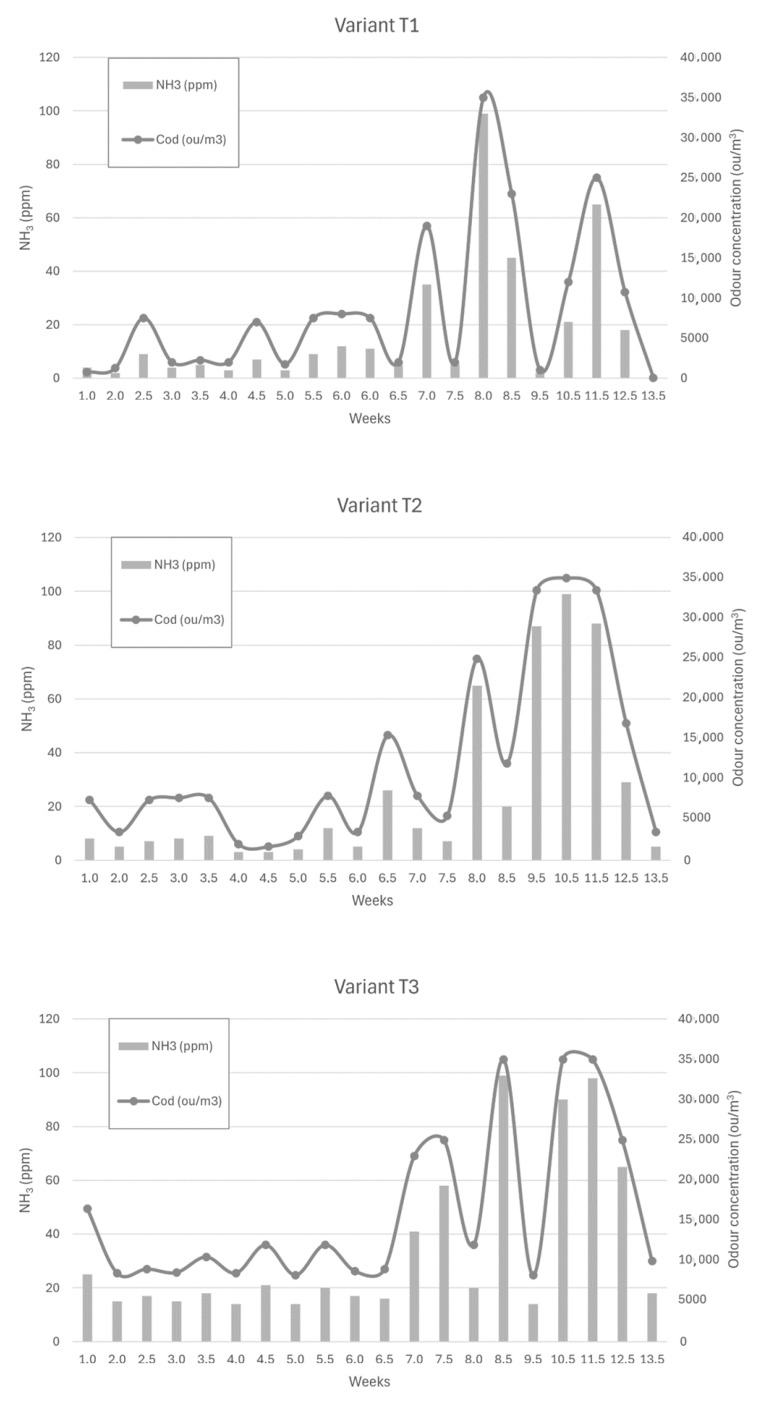
The dependence of ammonia and odour concentrations on the week of the biostabilisation process, with variants differing in preparation addition (preparation A, preparation B, and with no preparation addition) and the proportion of intermediate fraction. Variants T1–T3: 80% of waste and 20% of intermediate fraction.

**Figure 7:**
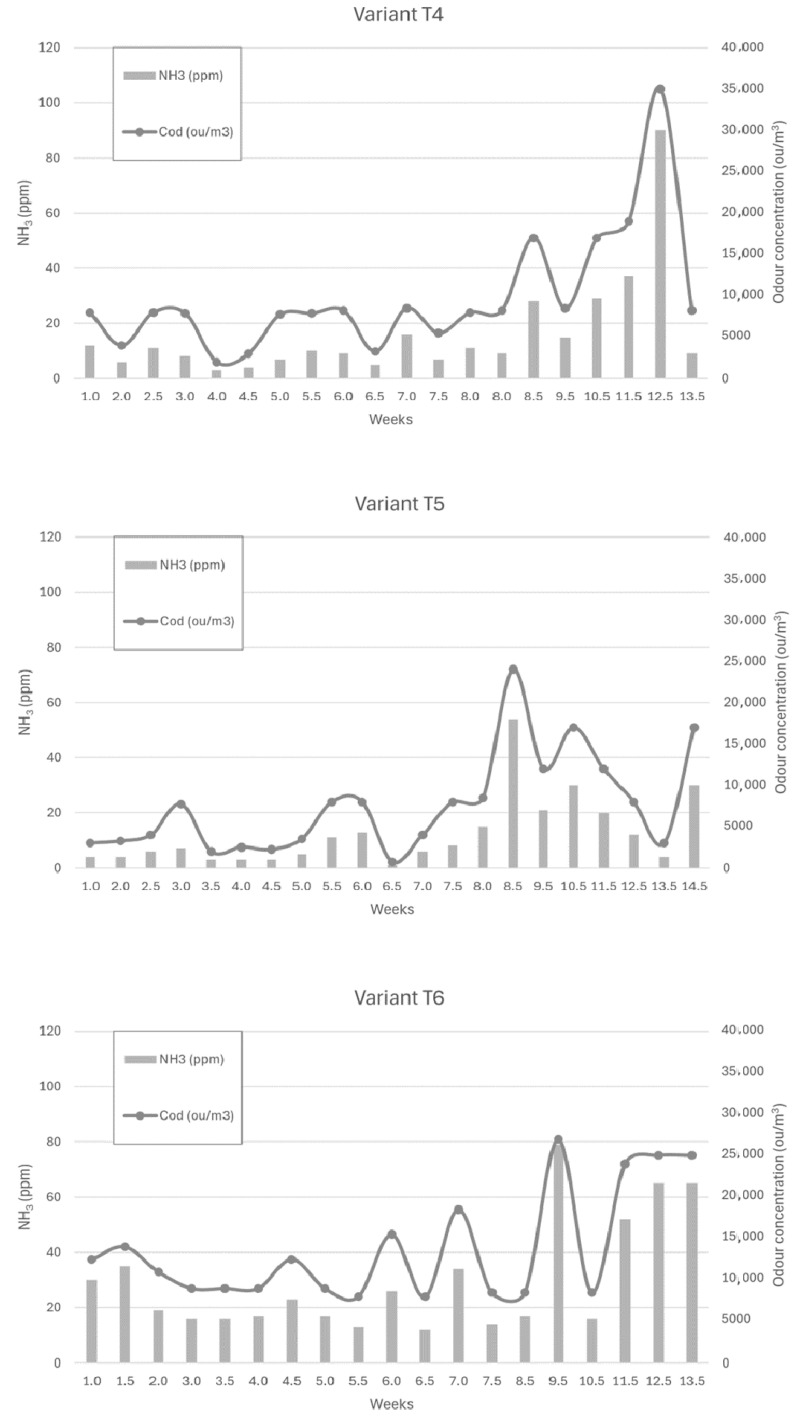
The dependence of ammonia and odour concentrations on the week of the biostabilisation process, with variants differing in preparation addition (preparation A, preparation B, and with no preparation addition) and the proportion of intermediate fraction. Variants T4–T6: 91% of waste and 9% of intermediate fraction.

**Figure 8:**
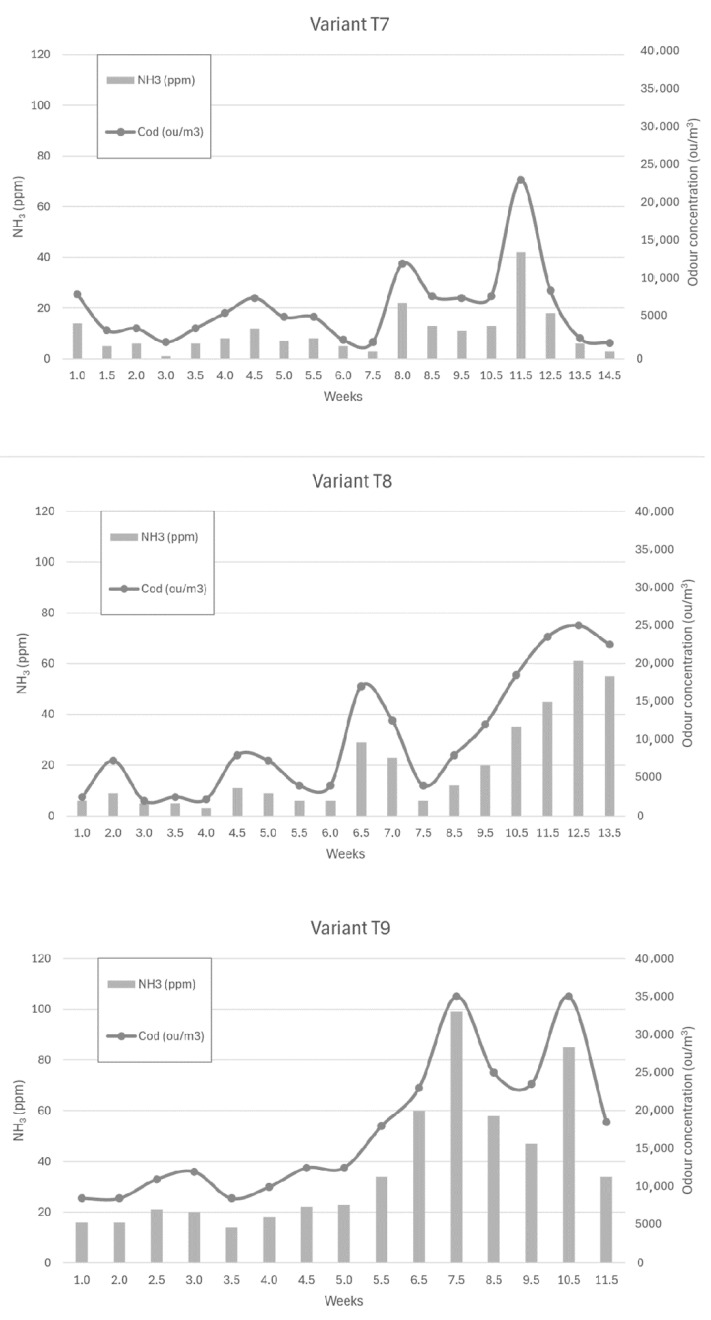
The dependence of ammonia and odour concentrations on the week of the biostabilisation process, with variants differing in preparation addition (preparation A, preparation B, and with no preparation addition) and the proportion of intermediate fraction. Variants T7–T9: without intermediate fraction.

Figure 6, Figure 7 and Figure 8 show the dependence of ammonia and odour concentrations depending on the week of biostabilisation, in the different variants differing in preparation addition (preparation A, preparation B, and with no preparation addition) and the proportion of intermediate fraction. Figure 6 shows variants T1–T3: 80% of waste and 20% of intermediate fraction, Figure 7 shows variants T4–T6: 91% of waste and 9% of intermediate fraction, and Figure 8 shows variants T7–T9: without intermediate fraction. 

#### 3.3.1. Preparation A (Variant T1)

Apart from three cases, the ammonia concentration at 10 cm depth did not exceed 40 ppm. In one case, at week 7, the ammonia concentration was 100 ppm; in one case, it was 65 ppm (11.5 weeks into the process); and in one case, it was 45 ppm (8.5 weeks). The increase in ammonia concentration occurred at week 7.5—from 5 ppm to 35 ppm—but this increase was most intense between weeks 7 and 8. The ammonia concentration was then 99 ppm, which dropped to 45 ppm at week 8.5. Another strong increase occurred between 10.5 weeks and 11.5 weeks; the ammonia concentration then increased from 21 ppm to 65 ppm. 

The results in this variant were consistent for the initial values of the composting process obtained by Biasoli et al. These authors obtained an odour concentration value of 750 ou/m^3^ for fresh compost, just after stacking (100% organics fraction). After 20 days, the aerated compost, after uncovering the gore-tex membrane, emitted an odour concentration equal to 1300 ou/m^3^ [49]. In the present study, for variant T1, the fresh compost showed an odour concentration equal to 750 ou/m^3^, while after two weeks, the concentration was 1250 ou/m^3^.

#### 3.3.2. Preparation 2 (Variant T2)

For the powder B variant, the increase in ammonia concentrations started at week 8 of the process—from a value of 7 ppm at week 7 to 65 ppm at week 8. The high ammonia concentrations continued until week 11.5, reaching 88 ppm, to drop to 29 ppm at week 12.5.

#### 3.3.3. Without Addition of Preparation (Variant T3)

For the no-addition variant, the increase in ammonia concentration occurred at week 7 of the process—from 16 ppm to 41 ppm. This was followed by the ammonia concentration reaching 99 ppm in 1.5 weeks (8.5 weeks of the process). At week 10.5 of the process, the ammonia concentration at a depth of 10 cm was 90 ppm, and at week 11 it was 99 ppm. In the following weeks, the ammonia concentration decreased, reaching 18 ppm in week 13.5 of the process. Scaglia et al. achieved similar values of odour concentration. These researchers studied the odour impact of the waste at a time ‘0’, after 4 weeks and after 13 weeks, obtaining 28,546 ou/m^3^, 4902 ou/m^3^, and 2569 ou/m^3^, respectively [7].

#### 3.3.4. Preparation A (Variant T4)

The ammonia concentration in the first six weeks of the trial varied between 5 ppm and 10 ppm. In week 8.5 it increased to 28 ppm, and in week 10.5 it was 29 ppm, to reach 37 ppm and 90 ppm in week 11.5 and week 12, respectively. After reaching a maximum, the ammonia concentration dropped to 13.5 ppm in week 9.

#### 3.3.5. Preparation 2 (Variant T5)

For the powder B variant, the ammonia concentration was stable up to week 8 (3–8 ppm), to increase to 54 ppm at week 8.5. At week 9.5 it was 21 ppm, and at week 10.5 it was 30 ppm, to successively decrease to 4 ppm at week 13.5 of the process.

#### 3.3.6. Without Addition of Preparation (Variant T6)

For the first 8.5 weeks of the trial, ammonia concentrations ranged from 12 to 35 ppm. In week 9.5, there was a sharp increase in ammonia concentration to 79 ppm, after which it dropped to 16 ppm. In the following weeks, the ammonia concentration increased, reaching 65 ppm in week 13.5 of the process. A similar trend was observed to that in the study by Defoer and van Langenhove, who observed the highest odour concentrations in the compost heaps in the first week of biostabilisation, the fifth and sixth weeks of biostabilisation, and the highest odour impact in the twelfth week [18]. The validity of the use of microorganism inoculation is confirmed in the study by Sekeran et al. [50] and Fan et al. As in this study, a decrease in odour performance of composting materials was observed already in the first three weeks compared to the control sample (without addition of preparation), where that decrease was observed after five weeks [51]. Khalil et al. noticed a decrease in the odour nuisance of the compost inoculated with effective organisms after 5 weeks, compared to the control sample, where the decrease was noted after 7 weeks of the process [52]. 

#### 3.3.7. Preparation A (Variant T7)

By week 7.5, ammonia concentrations ranged from 1 ppm to 12 ppm. By week 8, they had risen to 22 ppm and then, at week 11.5, to 42 ppm. In the following weeks, ammonia concentrations decreased to reach 3 ppm at week 14.5.

#### 3.3.8. Preparation 2 (Variant T8)

By week 6, ammonia concentrations ranged from 3 to 11 ppm, rising to 29 ppm by week 6.5. In the following weeks, the ammonia concentration decreased to 6 ppm at week 7.5. They then began to increase to a value of 61 ppm at week 12.5 of the process time.

#### 3.3.9. Without Addition of Preparation (Variant T9)

The ammonia concentration in the first weeks of the process ranged from 16 ppm to 23 ppm, rising to 34 ppm in week 5.5. Once this value was reached, the ammonia concentration started to increase rapidly to 60 ppm in week 6.5 of the process and 100 ppm in week 7.5 of the process. Then, after decreasing to 47 ppm (week 9.5), it increased to 85 ppm in week 10.5 before decreasing to 34 ppm in week 11.5 of the process.

#### 3.3.10. Summary of Comparison of Results by Week of a Study

For a proportion of 80% of waste and 20% of intermediate fraction, ammonia concentrations at 10 cm depth were on average 10–20 ppm higher each week for the variant without additive than for those with additive A (liquid) or B (powder). In the case of ammonia concentration—sampling at 10 cm depth—for a higher proportion of the intermediate fraction, lower concentrations were recorded for the variant with the addition of liquid A. Median of the results of ammonia concentration at 10 cm depth was, for the liquid, powder, and without addition of formulation, 7.00 ppm, 8.50 ppm, and 19.00 ppm. Of the ammonia concentration results in the heap inoculated with preparation A, 43% were in the 2–5 ppm range, compared with 30% for formulation B. Therefore, for formulation A, much larger variations in ammonia concentration at 10 cm depth were observed than for formulation B. 

For the ratio of 91% of waste and 9% of intermediate fraction, the ammonia concentration at 10 cm depth each week was on average 10–20 ppm higher for the variant without additive than for additive A or B. Lower concentrations were recorded for the variant with the addition of powder B. The median of the results was, for the liquid, powder, and without the addition of formulation, 9.50 ppm, 7.00 ppm, and 19.00 ppm. In the heap inoculated with preparation A, 40% results of the ammonia concentration were in the 6–10 ppm range (15%—2–5 ppm), compared with 19% for formulation B (33%—2–5 ppm). In this case, similar stability of ammonia concentration was noted with preparations A and B, as opposed to a ratio of 80% of waste and 20% of intermediate fraction.

Ammonia concentrations at a depth of 10 cm—in the variants without the contribution of the intermediate fraction—were, on average, 10–20 ppm higher each week in the variant without the addition of preparation than with the addition of preparation A or B. With formulation A, lower ammonia concentrations were determined than with formulation B. The median of the results was, for the liquid, powder, and without the addition of formulation, 4.00 ppm, 10.00 ppm, and 23.00 ppm. However, in this case, an increase in ammonia concentration was recorded for preparation B (powder) from week 7.5 of the process, lasting practically until the end of week 12.

A comparison of the concentrations of the tested compounds in the variants with preparation A or B shows that the values were similar. In the case of ammonia—sampling at a depth of 10 cm, a greater share of the intermediate fraction—lower concentrations were recorded for the variant with the addition of liquid A. However, a much greater variation in ammonia concentration was observed at a depth of 10 cm than for formulation B. 

In the case of formulation A, samples with a lower and higher proportion of the intermediate fraction had similar ammonia concentrations. In the variant with the higher proportion of intermediate fraction, a maximum ammonia concentration of 100 ppm was reached earlier (week 8) than in the lower proportion of intermediate fraction (week 12.5). The ammonia concentration was lowest in the variant without the intermediate fraction, ranging from 0 ppm to 40 ppm. 

For formulation B, much higher concentrations were found in the variant with a higher proportion of intermediate fraction than in the other variants. The lowest values were recorded for the variant with a lower proportion of the intermediate fraction. 

In the variants without added preparation, the highest concentrations were found in the variant with the highest proportion of the intermediate fraction. The values of ammonia concentrations for the variant with a smaller share of the intermediate fraction and without the intermediate fraction were comparable. 

To sum up, for ammonia—sampling at a depth of 10 cm—the lowest emissions during the whole composting process were recorded in the variants with additive A (liquid), with a 9% share of the intermediate fraction and without the intermediate fraction. It should be noted, however, that in this case, numerous ammonia concentration (and thus, odour concentration) peaks were observed. The concentration of this compound was much less stabilised than in preparation B (powder) in the variants with 20% and 9% share of intermediate fraction. 

## 4. Conclusions

Studies have shown that odour concentration is an important indicator of the maturity of composted waste. Olfactometric testing, particularly carried out in situ, can be a suitable method for monitoring the aerobic stabilisation process of waste, both as an individual method and in combination with methods currently in use. Using olfactometric testing in combination with ammonia testing and monitoring of the basic parameters of the composting process, the most effective technological solution was selected, both in terms of odour emissions—and thus, the safety of employees and residents of the surrounding areas—and the composition of the compost heaps.

This study showed the necessity for the use of a preparation; the concentrations of the tested compounds were significantly higher in the variants without both of the preparations B and A. Moreover, significantly lower stability of the tested concentrations and higher fluctuations were observed in the variants without the addition of the preparation. 

Studies on the odour impact of waste management facilities, in particular composting plants and mechanical–biological treatment plants, are of particular importance due to, among other things, their adverse impact on the environment and the related escalation of social conflicts.

As there are few studies in the literature on odour concentrations or odour emission levels from biological treatment plants, this study fits into the need to develop this topic.

In addition, the results obtained may make it possible to try to identify and clarify existing methodologies, especially in relation to the work on odour-related legislative guidelines. The present study may contribute to supplementing the above-mentioned methodologies with an additional indicator—odour concentration. This addition to the methodologies may allow the composting process to be monitored and controlled with an additional variable, complementary to the existing, costly analyses, and thus reduce the cost of the above-mentioned monitoring. In addition, it will allow for the optimisation of the composting process by selecting the optimum technological variant, considering both the proportion of fractions to be composted and the deodourisation method. This will enable better planning of the composting process, considering not only product quality, but also odour emissions. This solution will, therefore, minimise the impact of odours both on the health of the residents of the settlements located in the immediate vicinity of the composting plant and on the health of the personnel working at the plant.

Due to the high variability in emissions from week to week and from day to day, it seems essential to use sensors that continuously monitor the concentration of substances. Combined with odour concentration tests, this would give a complete picture of the operation of the composting plant, allowing peaks and fluctuations to be detected and linked to the technological operating regime of the plant. The issue of emissions during the screening and turning of waste may be of particular relevance. It is also reasonable to use more sensitive sensors to determine the continuous, in situ concentration of other odourogenic compounds, particularly VOCs.

## Figures and Tables

**Table 1:** List of analysed variants.

Share of the Intermediate Fraction	Addition of the Preparation		Without Addition
	A (Liquid)	B (Powder)	
	Variant		
80% of wastes (~452 m^3^) and 20% of intermediate fraction (~113 m^3^)	T1	T2	T3
91% of wastes (~514 m^3^) and 9% of intermediate fraction (~51 m^3^)	T4	T5	T6
No intermediate fraction	T7	T8	T9

## Data Availability

The original contributions presented in the study are included in the article, further inquiries can be directed to the corresponding author.
